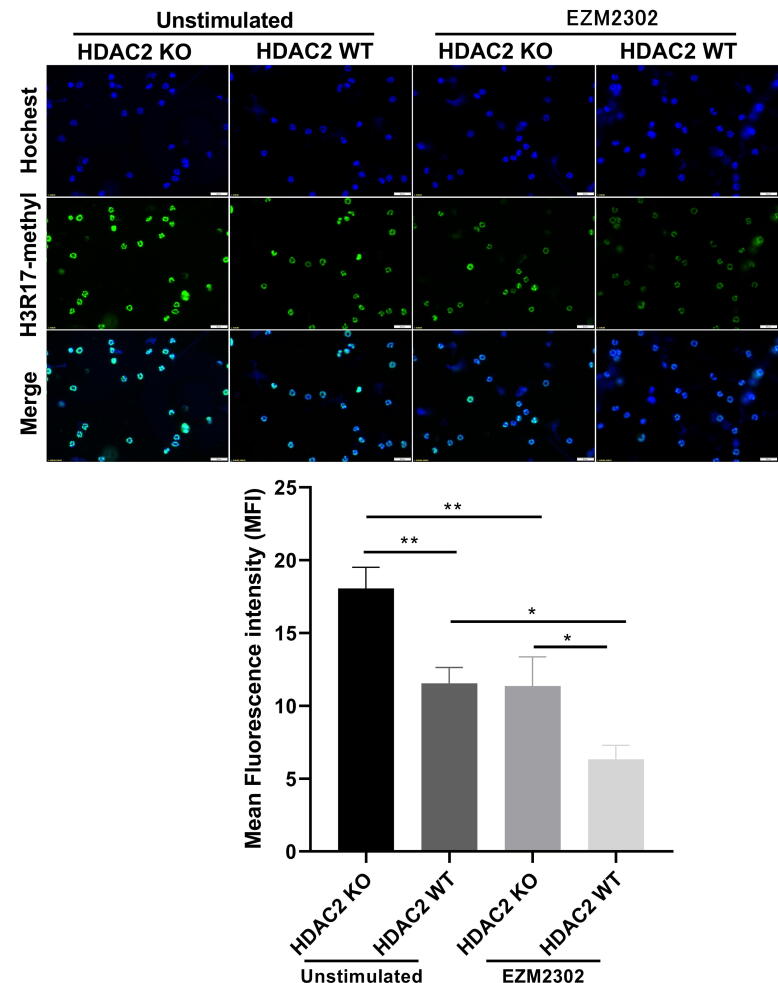# HDAC2 enhances the antimicrobial activity of neutrophils by promoting the formation of neutrophil extracellular traps (NETs) in sepsis

**DOI:** 10.1016/j.jare.2025.08.041

**Published:** 2025-08-22

**Authors:** Zhan Li, Wang Hu, Kaiyan Lv, Lumin Sui, Mu Yuan, Luoquan Ao, Quan Chen, Junxia Li, Lixing Tian, Zhengbi Liu, Sai Wang, Huaping Liang, Xiang Xu

**Affiliations:** a Department of Stem Cell & Regenerative Medicine, State Key Laboratory of Trauma and Chemical Poisoning, Daping Hospital, Army Medical University, Chongqing 400042, PR China; b Yunnan Key Laboratory of Stem Cell and Regenerative Medicine, Kunming Medical University, Kunming 650500, PR China; c Department of Laboratory Animal Center, Daping Hospital, Army Medical University, Chongqing 400042, PR China; d Department of Emergency Medicine, 953 Hospital of PLA Army, Shigatse Branch of Xinqiao Hospital, Army Medical University, Shigatse 857000, PR China; e Department of Wound Infection and Drug, State Key Laboratory of Trauma and Chemical Poisoning, Daping Hospital, Army Medical University, Chongqing 400042, PR China; f Chongqing Key Laboratory of Precision Diagnosis and Treatment for Kidney Diseases, Chongqing 400042, PR China

**Keywords:** Sepsis, Neutrophil extracellular traps, HDAC2, Cit-H3R17, Methy-H3R17, Acety-H3K18

## Abstract

**Introduction:**

Sepsis is characterized as a life-threatening organ dysfunction resulting from a dysregulated host response to infection, representing a critical clinical syndrome. Uncontrolled infection and inflammation are two main characteristics of sepsis. Neutrophil extracellular traps (NETs) is a major mechanism by which neutrophils resist pathogens invasion, but the mechanism of the NETs formation remain completely unclear.

**Objectives:**

This study aims to elucidate the role of Histone Deacetylase 2 (HDAC2) in NETs formation and its impact on antimicrobial and anti-inflammatory activities in sepsis.

**Methods:**

We employed flow cytometry, immunofluorescence and western blotting to assess NETs formation. The effects of HDAC2 on NETs and sepsis outcomes were investigated using an HDAC2 inhibitor and HDAC2 knockout mice in CLP-induced and LPS-induced sepsis models. Histone modifications in neutrophils were also analyzed to explore HDAC2’s regulatory mechanism.

**Results:**

HDAC2 was found to be highly expressed in patients and mice with sepsis. While HDAC2 knockout or inhibition reduced inflammation and improved organ function in non-infectious sepsis, it decreased survival in infectious sepsis. In addition, knockout or inhibition of HDAC2 significantly reduced the NETs formation, and impaired the antimicrobial activities against *E. coli* infection in mice. Mechanistically, HDAC2 indirectly promoted H3R17 citrullination to induce the NETs formation through down-regulating H3K18 acetylation and interactively inhibiting CARM1-mediated H3R17 methylation in neutrophils. Furthermore, a dual inhibition strategy targeting HDAC2 and CARM1 not only suppressed inflammation, prevented from multiple organ dysfunction, but also enhanced the antimicrobial activities, which finally improved survival rate of mice with sepsis induced by cecal ligation and puncture (CLP).

**Conclusion:**

Our findings reveal a novel role for HDAC2 in modulating anti-infectious immunity through NETs formation during sepsis. This study provides a potential therapeutic strategy for sepsis by enhancing both antimicrobial and anti-inflammatory responses, offering a promising approach to preserve organ function and improve survival.

## Highlights

• HDAC2 highly expressed in septic patients and mice.
• HDAC2 loss curbs sterile sepsis but worsens infectious sepsis.
• HDAC2 drives NET formation via histone modification.
• Dual HDAC2/CARM1 inhibition boosts antimicrobial and anti-inflammatory efficacy.

## Graphical abstract

HDAC2 plays a crucial role in the NETs formation by directly regulating histone acetylation modifications, and interactively alternating histone methylation and citrullination modification, which is crucial for antimicrobial defense and inflammatory responses by modulating histone modifications in neutrophils. This figure was created by Figdraw.

**Figure:**
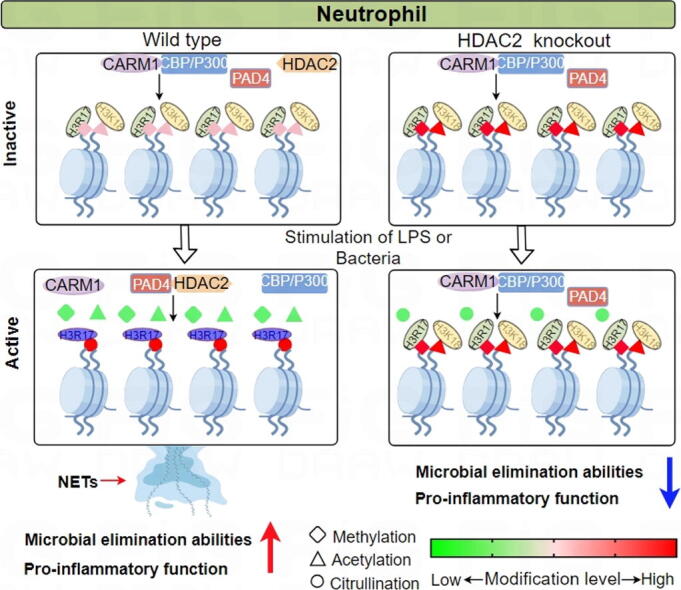

## Introduction

Sepsis is characterized as a complex syndrome involving systemic inflammation, immune system dysregulation, coagulation cascade disruption, and endothelial dysfunction, all of which are triggered by the presence of invading pathogens [1,2]. Sepsis is typically initiated by severe infections caused by pathogenic microorganisms [3]. Currently, the management of sepsis primarily relies on antibiotic therapy. However, the widespread use of antibiotics has resulted in increased bacterial resistance, which complicates subsequent treatment efforts [3]. Despite significant advancements in critical care, including the deployment of appropriate antibiotic regimens and resuscitative measures, the incidence of sepsis worldwide and the mortality rates associated with severe sepsis have remained strikingly high, indicating the need for further research and innovative therapeutic strategies. Furthermore, when a patient advances to septic shock, the elimination of pathogens by antibiotic therapy can trigger the release of additional toxic substances. This release may exacerbate the patient's clinical condition, leading to a deterioration in their health status [4]. Therefore, developing a new antimicrobial strategy is critically important for the clinical management of sepsis, as it has the potential to significantly improve therapeutic outcomes and patient survival rates.

Neutrophils, as a principal component of the innate immune system, typically act as the primary cellular defense against pathogen invasion. They are instrumental in mediating anti-infectious immunity and inflammation during the initial phase of sepsis, thereby playing a pivotal role in the body's early response to infection [[5], [6], [7]]. One of them, the formation of neutrophil extracellular traps (NETs) represents a significant mechanism through which neutrophils exert their antimicrobial effects, ensnaring and neutralizing a broad spectrum of pathogens [5,8]. During the initial phase of numerous infectious diseases, neutrophils, upon encountering pathogen-associated molecular patterns (PAMPs) such as LPS, promptly initiate the NETs formation and eliminate pathogens in a timely and rapid manner [[8], [9], [10]]. Recent research indicates that neutrophil extracellular traps (NETs) serve as innate defense barriers composed of neutrophil elastase (NE), myeloperoxidase (MPO) and histones, and NETs are formed by neutrophils in response to various stimuli, such as bacteria, lipopolysaccharide (LPS) and phorbol myristate (PMA) [9,11,12]. NETs formation depends on histone citrullination which is catalyzed by peptidyl arginine deiminase 4 (PAD4) [13,14]. Although some researches have demonstrated that a strong correlation between NETs formation and the generation of reactive oxygen species (ROS) as well as PAD4-catalyzed histone citrullination [10,13,[15], [16], [17], [18]], it is unclear whether other histone modifications, such as histone acetylation and methylation, are involved in regulating the formation of NETs. Consequently, further researches on the mechanism underlying NETs formation are of great significance for the clinical treatment of sepsis.

Methylation, acetylation of arginine and lysine residues in histones are the main epigenetic forms of the regulation of target genes expression. These epigenetic modifications of histones are regulated by a series of enzymes. One of them, HDAC2 belongs to class I of the histone deacetylase (HDAC) family. It functions as a zinc ion-dependent metalloprotease that specifically removes acetyl groups from lysine residues in the N-terminal region of core histones (H2A, H2B, H3, and H4). By remodeling the chromosome structure, HDAC2 is instrumental to the regulation of transcriptional expression of target genes. Recently, some studies have shown that HDAC2 is involved in the regulation of the natural immune response, and plays an important role in the development of inflammatory diseases such as sepsis; the deficiency or inhibition of HDAC2 significantly enhances the survival rate of septic mice by weakening the inflammatory responses [[19], [20], [21], [22]]. Although HDAC2 may regulate inflammatory responses, it remains unclear whether HDAC2 is involved in the regulation of anti-infectious immunity.

As mentioned above, the formation of NETs depends on histone citrullination catalyzed by PAD4. In addition, PAD4 down-regulates Arg methylation, including histone H3 Arg17 site methylated by coactivator associated arginine methyltransferase 1 (CARM1), by converting methyl-Arg to citrulline and releasing methylamine [13]. The evidence has demonstrated that histone acetyl transferases CBP and P300 (CBP/P300) up-regulates H3K18 and H3K23 acetylation which can enhance histone H3R17 methylation by increasing the methyltransferase activity of CARM1 [22]. Additionally, H3K18, H3K14 and H3K56 in some gene promoter regions were found to be targets of HDAC1/2 [23,24]. Based on these findings, we hypothesize that HDAC2 may indirectly enhance the formation of NETs by inhibiting histone H3K18 acetylation and histone H3 Arg17 methylation, which can ultimately enhance histone H3 Arg17 citrullination. In the present study, we have made several significant discoveries. First, we have identified that HDAC2 exerts antimicrobial activity through promoting the formation of NETs. Second, we have elucidated the underlying mechanism by which HDAC2 enhances NETs formation. Specifically, HDAC2 inhibits histone H3K18 acetylation and histone H3R17 methylation, thereby up-regulating histone H3R17 citrullination. Third, we have innovatively proposed an effective strategy for simultaneously inhibiting CARM1 and HDAC2. This strategy not only inhibits inflammatory responses but also maintains anti-infectious abilities in septic mice. Collectively, our research has broadened the current molecular understanding of anti-infectious immunity via NETs formation in sepsis and may provide new insights for the development of therapeutic interventions targeting sepsis-related infections.

## Material and methods

### Microarray analysis of gene expression datasets for sepsis

Sepsis high-throughput gene chips GSE95233 were screened from the Gene Expression Omnibus (GEO) database of the National Center for Biotechnology Information (https://www.ncbi.nlm.nih.gov/geo). GSE95233 datasets for healthy and sepsis samples were obtained from the GEO repository. A total of 22 healthy samples and 102 sepsis samples were included in the prognostic analysis. The expression of HDAC2 mRNA was analyzed.

### Real-time quantitative PCR

Peripheral blood leukocytes were isolated from peripheral blood of CLP (or 10 mg/kg LPS)-induced septic mice for 6 h. Total RNA was meticulously isolated using the FastPure Cell/Tissue Total RNA Isolation Kit V2 (RC112-01, Vazyme, China), adhering to the manufacturer's protocol. Subsequent cDNA synthesis was executed with the HiScript®III RT SuperMix for qPCR (R323-01, Vazyme, China), following the provided guidelines. Quantitative real-time PCR (qRT-PCR) was performed utilizing the ChamQ Universal SYBR qPCR Master Mix (Q711-02, Vazyme, China). The β-actin gene served as an internal control to standardize the expression levels. The mRNA expression levels of HDAC2 were specifically detected. The sequences of the primers were used as follows: HDAC2 forward primer 5′-TGTCAATTGGGCTGGAGGAC-3′ and reverse primer 5′-TCGAGGATGGCAAGCACAAT-3′, and β-actin forward primer 5′-CCCAGGCATTGCTGACAGG-3′ and reverse primer 5′-TGGAAGGTGGACAGTGAGGC-3.

### Animals experiments

C57BL6J mice (6–8 weeks old, male) were supplied by SI PEI FU Co. Ltd. (Beijing, China). HDAC2 KO mice (6–8 weeks old, male) were supplied by Jackson Laboratory. Army Medical University and institutional review board of Daping Hospital approved all the procedures. For mouse models of CLP-induced sepsis with HDAC2 inhibition, the animals received an intraperitoneal injection of SAHA (25 mg/kg; MCE, HY-10221) after 0.5 h CLP modeling; For mouse models of LPS-induced sepsis with HDAC2 inhibition, the mice were injected intraperitoneally with LPS (10 mg/kg; Sigma, L4130, derived from *E. coli* O111:B4) after 1 h injection of SAHA. Negative control (NC) was typically the vehicle control (saline injection). For mouse models of dual strategy for HDAC2 and CARM1 inhibition, the mice were injected intraperitoneally with EZM2302 (25 mg/kg; TargetMOI, T5605, China) in CLP mice for 18 h prior to a treatment with SAHA. All the mice were monitored for survival. Survival was based on the number of hours from the injection SAHA or LPS to the hour of animals were to be euthanized. The results were analyzed using GraphPad Prism 8.0 soft-ware. Mice were anesthetized and sacrificed.

### Bacterial culture test

*E. coli*-GFP strains were derived from ATCC 25922. HDAC2 WT and HDAC2 KO mice were administrated with *E. coli-*GFP (5 × 10^7^) by intravenous Injection. Blood (20 μl) was taken from the tail vein of the mice. Fresh tissue samples were weighed and PBS was added to grind it in a 1 (g):9 (ml) ratio. After grinding, the supernatant was centrifuged (4 °C, 3000 rpm for 10 min) and absorbed. This supernatant was serially diluted 10 times and 20 μl liquid was applied to pretreated solid Mueller-Hinton agar (MHA) medium, and incubated at 37 °C for 20 h. The resulting colonies were enumerated and photographed.

### Mice neutrophil isolation

Mice neutrophils were extracted from the peripheral blood of HDAC2 KO or HDAC2 WT mice and diluted with the 15 mM ethylenediaminetetraacetic acid (EDTA) anticoagulation buffer containing 0.5 % BSA/PBS solution. 4 ml of Histopaque-1119 (Sigma-Aldrich) and Histopaque-1077 (Sigma-Aldrich) were successively added to 15 ml conical centrifuge tubes, and then the cell suspension was added to the upper layer of Histopaque-1077 and centrifuged at 800 g for 30 min. As a result, neutrophils were subsequently harvested from the interphase between the Histopaque-1119 and Histopaque-1077 layers.

### Western blot

Mice neutrophils were isolated from whole blood and bone marrow using the gradient density centrifugation method, which involved the application of Histopaque solutions with densities of 1077 and 1119. Cells were resuspended at a density of 2.4 × 10^6^/ml. And they were pretreated with or without LPS (50 μg/ml) or SAHA (5 μM) for 2 h. During treatment, cells were maintained in a 37 °C incubator with a 5 % CO_2_ atmosphere. Following stimulation, cells were centrifuged, and the pellets were treated on ice with lysis buffer and frozen overnight. On the subsequent day, the lysates were thawed on ice and sonicated for 5 × 30-s bursts at 4 °C. Lysates were separated by SDS-PAGE. Western blotting was performed to detect citrullination, acetylation and methylation using the anti-Histone H3 (citrulline R2 + R8 + R17) antibody (abcam, ab5103, 1 μg/ml), anti-Histone H3 (citrulline R17) antibody (abcam, ab219407, 5 μg/ml), anti-Histone H3 (acetyl K18) antibody (abcam, ab177870, 5 μg/ml) and anti-Histone H3 (mono methyl R17) antibody (abcam, ab177222, 5 μg/ml) monoclonal antibody. Anti-Histone H3 (abcam, ab1791, 1 μg/ml) was used as a loading control, followed by HRP-conjugated goat anti-rabbit IgG secondary antibody (abcam, ab97051, 0.1 μg/ml).

### Neutrophils phagocytosis assay of *E. coli*-GFP in vitro

Neutrophils were isolated from peripheral blood of HDAC2 KO or HDAC2 WT mice, the phagocytosis of *E. coli*-GFP bacteria by blood neutrophils was investigated. The cell concentrations of both neutrophils and *E. coli*-GFP were adjusted to equal levels, with a ratio of neutrophil (1 × 10^5^cells/ml) to *E. coli*-GFP bacteria set at 1:20. The neutrophils were incubated in 1640 medium supplemented with 10 % FBS for 2 h at 37 °C. The proportion of FITC or GFP neutrophils were measured using flow cytometric analysis. Subsequently, the cells underwent a dual wash with phosphate-buffered saline (PBS), followed by centrifugation at a speed of 1000 revolutions per minute (rpm) for 5 min, resuspended in 2 ml PBS, and centrifuged again at 10000 rpm for rapid freezing. The cells were resuspended once more, and 20 μl of cell lysate was plated onto agar plates. After 20 h of incubation, the number of colonies was recorded.

### Immunofluorescence assay

15 mm diameter Scientific Round Cover Glass (801007, NEST, China) was incubated with 0.1 mg/ml Poly-d-lysine (E607014, Sangon Biotech, China) for 30 min at room temperature, then rinse the surface with sterile distilled water and dry 1 h at room temperature before seeding cells. Neutrophils were extracted from the peripheral blood of HDAC2 WT and HDAC2 KO mice and diluted to 2 × 10^5^/ml. Place scientific round Cover Glass into a 24-well plate and add neutrophils into it for 30 min. Subsequently, the cells were challenged with LPS (50 μg/ml) for 2 h at 37 °C. The cells were fixed using a 2 % paraformaldehyde (PFA) solution for 45 min at room temperature, permeabilized with PBS containing 2 % Triton X-100 for 10 min, and washed with PBS. Cells were washed with PBS and blocked in PBS containing 2 % BSA for 30mins at 4 °C. H3R17 citrullination, H3K18 acetylation and H3R17 methylation were measured using anti-Histone H3 (citrulline R17) antibody (abcam, ab219407, 5 μg/ml), anti-Histone H3 (acetyl K18) antibody (abcam, ab177870, 5 μg/ml) and anti-Histone H3 (mono methyl R17) antibody (abcam, ab177222, 5 μg/ml) monoclonal antibody, respectively. MPO imaging assays for detecting NETs formation are made in the same way as described above. Anti-mouse MPO (abcam, ab221847, 5 μg/ml) was used. Then we cultured them using a secondary Alexa Fluor 488 goat anti-rabbit IgG (abcam, ab150077, 1 μg/ml) with parallel Hoechst 33342 staining (Beyotime Biotech, C1026, 10 μg/ml).

### Flow cytometry analysis

Neutrophils were extracted from the peripheral blood of HDAC2 KO or HDAC2 WT mice, and 1 × 10^5^ cells/mL neutrophils were seeded into a 12-well plate and stimulated with 50 μg/mL LPS for 2 h, then collected into 1.5 ml centrifuge tubes, and cells were added to 0.5 ml of 1× Foxp3 fixation/membrane-breaking buffer (eBioscience™, 00-5523-00) per tube and incubated at room temperature in the dark for 30 min. The cells were washed with PBS and stained with anti-Histone H3 (citrulline R2 + R8 + R17) antibody (abcam, ab5103, 1 μg/mL) for 30 min on ice. Finally, cells were washed again with PBS and incubated with anti-rabbit IgG-Alexa Fluor 488 (CST, 4412S, 10 μg/mL) in PBS. After washing with PBS, the samples were analyzed by flow cytometer (ACEA NovoCyte D2060R, China).

### Histopathologic evaluation of tissues

The mice were treated with EZM2302 (25 mg/kg; TargetMOI, T5605, China) two hours prior to cecal ligation and puncture (CLP), and SAHA (25 mg/kg; MCE, HY-10221) was administered after CLP surgery. The fresh tissue samples were fixed in 4 % Paraformaldehyde (PFA) solution for 24 h, followed by a series of processes including dehydration, clarification, wax impregnation, and embedding in paraffin. 5-μm sections were cut from the histological samples and stained with hematoxylin-eosin. Furthermore, the cell apoptosis in lungs, kidneys and livers was stained with cleaved-caspase 3. Then, the stained tissue sections were then examined under a light microscope to evaluate histopathological changes in the lung, kidney and liver of mice.

### Serum detection

Concentrations of interleukin-1 beta (IL-1β), interleukin-6 (IL-6), histone deacetylase 2 (HDAC2), PCT, CRP and myeloperoxidase (MPO) in mouse serum were quantified using their respective enzyme-linked immunosorbent assay (ELISA) kits, following the protocols provided by the manufacturer (Boster Biological Technology, Wuhan, China). Additionally, the serum levels of alanine aminotransferase (ALT), aspartate aminotransferase (AST), amylase (AMS), creatinine (Crea), and urea were measured using an automated chemistry analyzer (AU5800, Beckman Coulter, USA).

### Statistical analysis

Data analysis was conducted using GraphPad Prism 8.0 (GraphPad Software, La Jolla, CA, USA). The results are depicted as the mean values accompanied by their standard error of the mean (SEM) to indicate variability. For statistical comparisons between two groups, the Student’s *t*-test was employed. When assessing differences among multiple groups, a one-way analysis of variance (ANOVA) was utilized, followed by post-hoc tests using the two-tailed Student’s *t*-test where appropriate. The survival curves were constructed employing the Kaplan-Meier estimation technique, and the differences between the curves were statistically assessed using the log-rank test. The threshold for statistical significance was established at P < 0.05.

## Results

### HDAC2’s role in sepsis pathogenesis

**Fig. 1:**
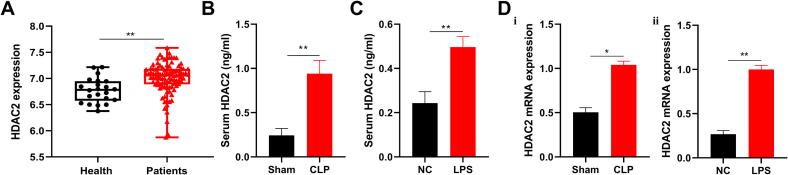
HDAC2 is highly expressed in sepsis. (A) Analysis of HDAC2 expression in GEO. HDAC2 expression was analyzed using the GSE95233 dataset, including 22 healthy controls and a total of 102 patients with sepsis. (B)The level of HDAC2 in serum was detected in the CLP-induced septic mice. Blood was collected from mouse orbits, and the level of HDAC2 was measured by Elisa. (C) The level of HDAC2 in serum was detected in the LPS (10 mg/kg)-induced septic mice. Blood was collected from mouse orbits, and the level of HDAC2 was measured by Elisa. (D) The HDAC2 mRNA expression in the peripheral blood leukocytes of (i) CLP-induced septic mice and (ii) 10 mg/kg LPS-induced septic mice. Data are presented as the means ± SEM. of independent experiments. *P < 0.05, **P < 0.01 (two-tailed Student’s *t*-test). n = 5 mice per group.

To investigate the role of HDAC2 in sepsis, we first analyzed the GSE95233 dataset, and found that HDAC2 is significantly highly expressed in patients with sepsis (Fig. 1A). Then, we detected the level of HDAC2 in CLP-induced (infectious) and LPS-induced (non-infectious) septic mice. The results showed that HDAC2 was significantly elevated in the two septic mice models (Fig. 1B–D). Therefore, it suggested that HDAC2 may be involved in the development of sepsis.

**Fig. 2:**
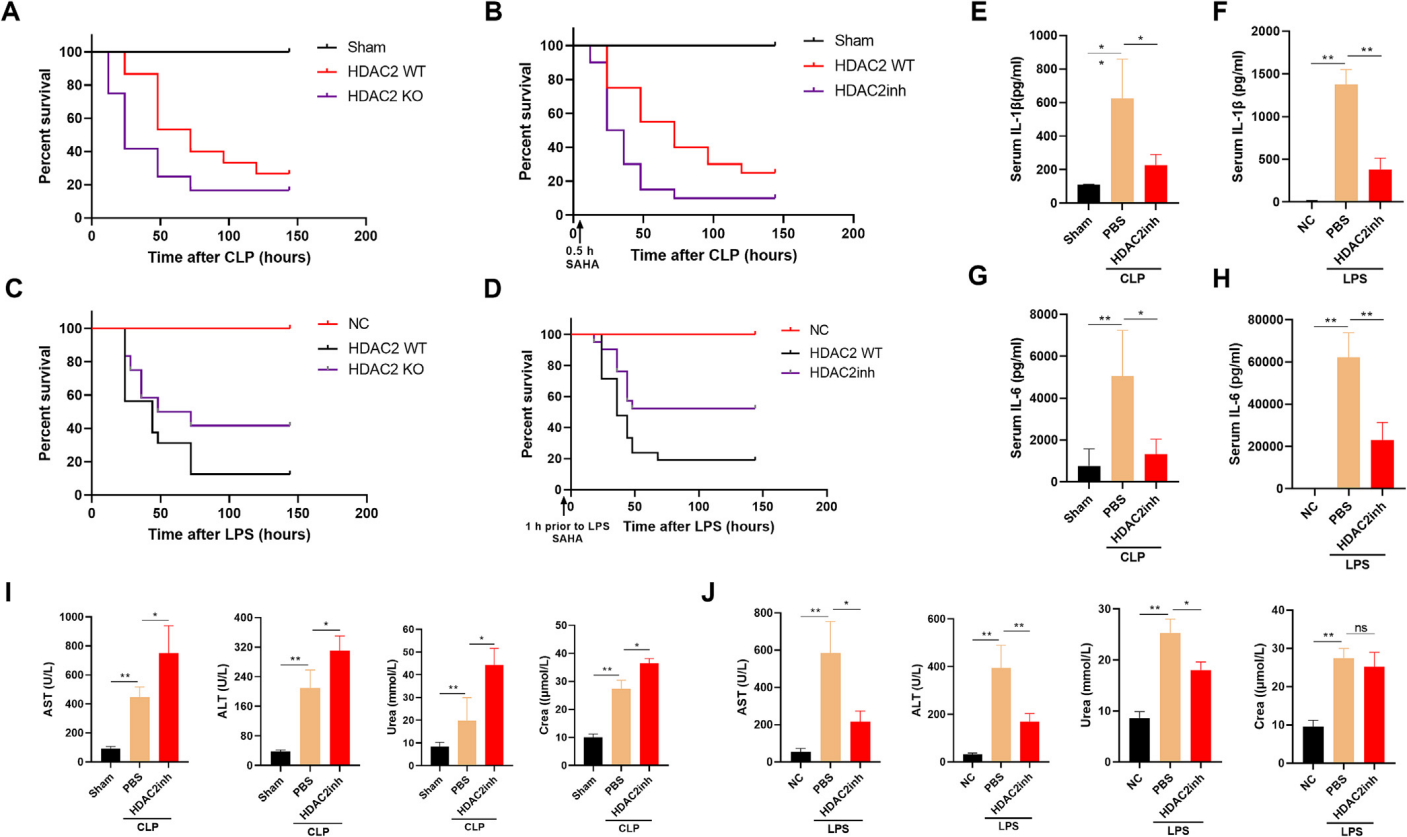
HDAC2 deficiency or inhibition compromises the antimicrobial efficacy, but improves anti-inflammatory responses in the mice with sepsis. (A-D) Effects of the HDAC2-knockout or inhibition on the survival of mice with CLP-induced or LPS-induced sepsis. Kaplan-Meier curves for the HDAC2-knockout or inhibition by SAHA-treated mice with or CLP-induced or LPS-induced sepsis (n = 20 mice). (E-H) Serum IL-6 and IL-1β levels were determined in the sham or CLP-induced or LPS-induced septic mice treated with or without SAHA (25 mg/kg). These inflammatory parameters were measured by ELISA. Data are shown as mean ± SEM. *p < 0.05; n = 5 mice per group. (I and J) Serum AST, ALT, Urea and Crea levels were measured in sham or CLP-induced or LPS-induced septic mice treated with or without SAHA. Blood was collected by orbital sampling, and these biochemical parameters were measured by the Automatic Biochemistry Analyzer. Data are shown as mean ± SEM. **p < 0.01; n = 5 mice per group.

To further elucidate the role of HDAC2 besides the regulation of inflammatory responses in sepsis, two septic mice models including LPS-induced sepsis and CLP-induced sepsis were constructed. We discovered that HDAC2 knockout or inhibition significantly reduced the level of inflammatory factors such as IL-6 and IL-1β (Fig. 2E and G), but the survival rate (Fig. 2 A and B) and multiple organ dysfunction including blood biochemical indexes such as ALT, AST, Urea and Crea (Fig. 2I) were not improved in CLP-induced (infectious) sepsis. However, in LPS-induced (non-infectious) septic mice, the level of inflammatory factors such as IL-6 and IL-1β was also decreased (Fig. 2 F and H), besides the survival rate (Fig. 2 C and D) and multiple organ dysfunction including blood biochemical indexes such as ALT, AST, Urea and Crea (Fig. 2 J) were improved following HDAC2 deficiency or inhibition. These results suggest that HDAC2 may play other unknown functions in addition to its involvement in the regulation of inflammatory responses in sepsis.

### HDAC2 enhances the antimicrobial activities against *E. coli* in vivo

**Fig. 3:**
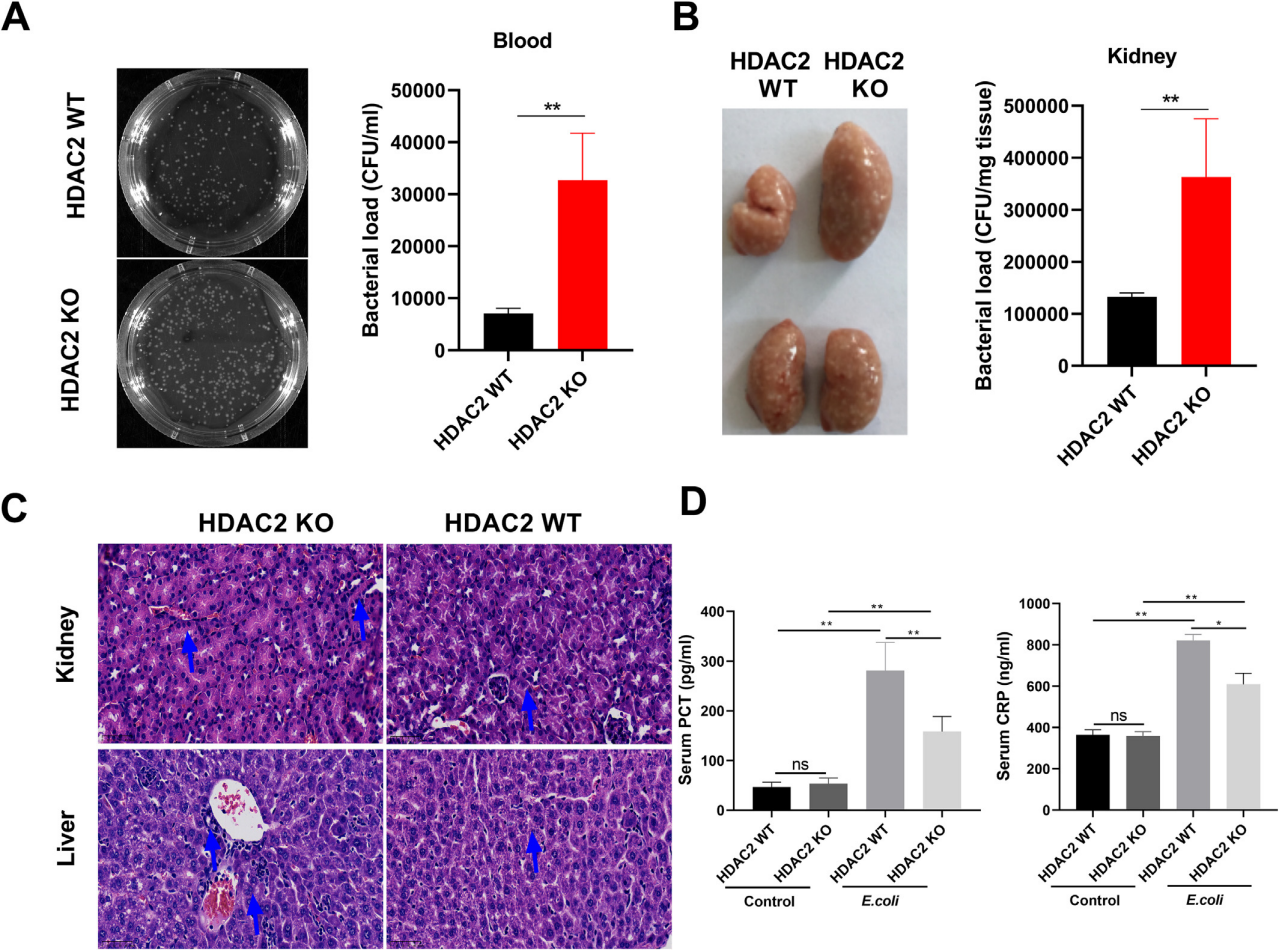
HDAC2 enhances antimicrobial activities against *E.coli* in mice. (A) Bacterial loads in the blood were calculated in HDAC2-knockout and wild type mice. (A)The picture of the bacterial clones was taken by BIO-RAD ChemiDoc“MP Imaging System. (B)The number of colony-forming units (CFU) in the blood of mice was calculated. HDAC2 WT and HDAC2 KO mice were administrated with *E. coli-*GFP (5 × 10^7^) by intravenous Injection. Blood (20 μl) was taken from the tail vein of the mice, applied to the pretreated solid MHA medium, and incubated at 37 °C for 20 h. Count the colonies on the culture medium and take pictures. The experiments were performed in quintuplicate, data are presented as the means ± SEM. of independent experiments. *P < 0.05, **P < 0.01 (two-tailed Student’s *t*-test). (B) Bacterial loads in the kidneys were calculated in HDAC2-knockout and wild type mice. The picture of kidneys from HDAC2 WT and HDAC2 KO mice were taken. The number of colony-forming units (CFU) in the kidneys of mice was calculated. The kidneys from HDAC2 KO and HDAC2 WT mice were weighed, ground, spread on the MHA plates, incubated for 16–24 h at 37 °C, and then bacterial clones were counted. The experiments were performed in quintuplicate, data are presented as the means ± SEM. of independent experiments. *P < 0.05, **P < 0.01 (two-tailed Student’s *t*-test). n = 5 mice per group. (C) H&E staining of livers and kidneys. The arrows indicate damage section of tissue. (D) Serum CRP and PCT levels of mice with *E. coli-*GFP bacteremia at 24 h. The CRP and PCT were measured by ELISA. Data are shown as mean ± SEM. *p < 0.05; n = 5 mice per group. ns, not significant.

To further analyze the function of HDAC2 in bacterial killing, we treated HDAC2 *KO* and *WT* mice with *E. coli* and assessed the contribution of HDAC2 in the context of bacterial infection. The results showed that compared with HDAC2 WT mice, HDAC2 KO mice exhibited significant swelling and a notable increase in blood and renal bacterial loads (Fig. 3A and B). Following intravenous injection of *E. coli* to HDAC2 WT and HDAC2 KO mice, HDAC2 KO mice demonstrated a significantly damage section of livers and kidneys, and lower serum concentrations of procalcitonin (PCT) and C-reactive protein (CRP) as diagnostic biomarkers for bacterial infection detection compared with WT mice (Fig. 3C and D). Therefore, these results further demonstrated that HDAC2 may play a key role in the regulation of anti-infectious immunity in vivo.

### HDAC2 is required for the NETs formation

**Fig. 4:**
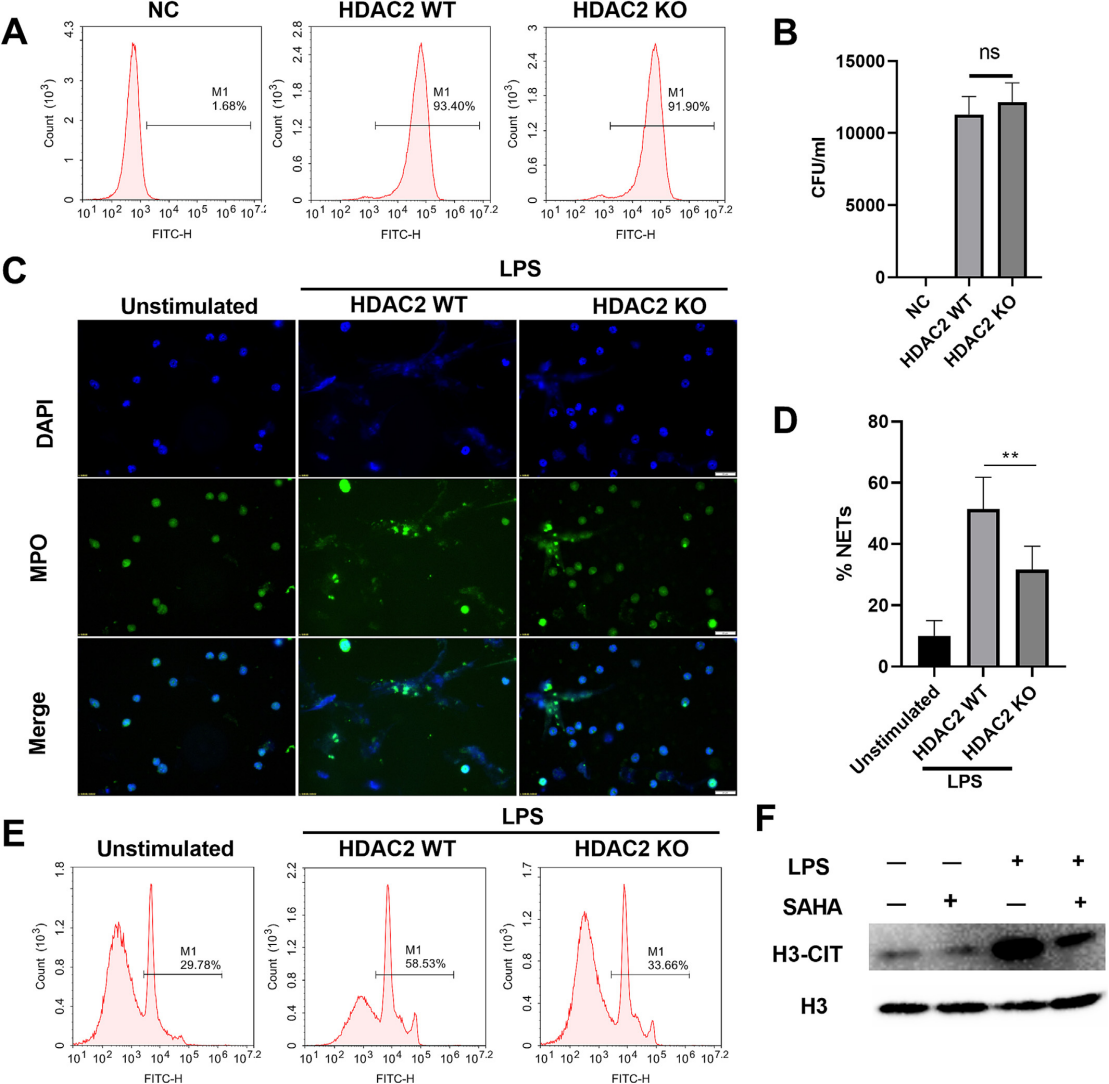
HDAC2 is required for NET formation. (A and B) Phagocytic uptake and intracellular killing in neutrophils were detected by flow-based assay and colon-formation assay. (A) Phagocytic uptake in neutrophils was detected by flow-based assay. Neutrophils were isolated from peripheral blood of HDAC2 KO or HDAC2 WT mice, and they were co-incubated with *E. coli*-GFP for two hours. (B) Intracellular killing in neutrophils was detected by colon-formation assay. Blood was taken from the tail vein of the mice, then applied to the pretreated solid MHA medium and incubated at 37 °C for 24 h. The number of bacterial clones was calculated. n = 5 mice per group. (C-D) NETs were detected in the neutrophils from peripheral blood of HDAC2 KO or HDAC2 WT mice by immunofluorescence. Representative data showing MPO-positive cells defined as positive NETs. Data are presented as the means ± SEM. of independent experiments. *P < 0.05, **P < 0.01 (two-tailed Student’s *t*-test). (E) NETs were detected in the neutrophils from peripheral blood of HDAC2 KO or HDAC2 WT mice by a flow cytometry-based assay. Representative flow data showing positive cells in global Histone H3 citrullination of H3R2, H3R8 or H3R17 sites, defined as positive NETs. (F) NETs markers were detected by western blotting in the neutrophils from HDAC2 WT mice after stimulation with LPS (50 μg/ml) and SAHA (5 μM). Representative data showing H3-Cit expression defined as NETs. The H3 protein was used for western blot loading controls.

Previous studies have shown that equipped with a full arsenal of antimicrobial proteins at their disposal, neutrophils have a well described role in phagocytic uptake and intracellular killing [8,9]. To test whether HDAC2 affects bacterial phagocytic uptake and intracellular killing of neutrophils, neutrophils were isolated from the peripheral blood of HDAC2 KO or WT mice, and incubated with *E. coli*-GFP for 2 h. The results showed that the phagocytic uptake and killing capabilities of neutrophils had no significant differences between HDAC2 KO and WT neutrophils (Fig. 4A and B). Hence, it suggested that HDAC2 did not exert the antimicrobial activities through regulating the phagocytic uptake and intracellular killing activities of neutrophils.

In order to further investigate the role of HDAC2 in anti-infectious immunity, we detected the NETs formation, and the results showed that the level of MPO, a NETs marker, was significantly increased after LPS treatment in wild type neutrophils, but the increases were significantly inhibited in HDAC2-knockout neutrophils (Fig. 4C and D). Similarly, After LPS treatment, the level of citrullination in H3R2, H3R8 or H3R17 sites, a NETs marker, was also significantly increased, but the increases were significantly inhibited by HDAC2 knockout or inhibition (Fig. 4E and F). To summarize, these findings have suggested that HDAC2 is involved in the regulation of NETs formation.

### HDAC2’s regulation role of NETs formation via histone modifications

**Fig. 5:**
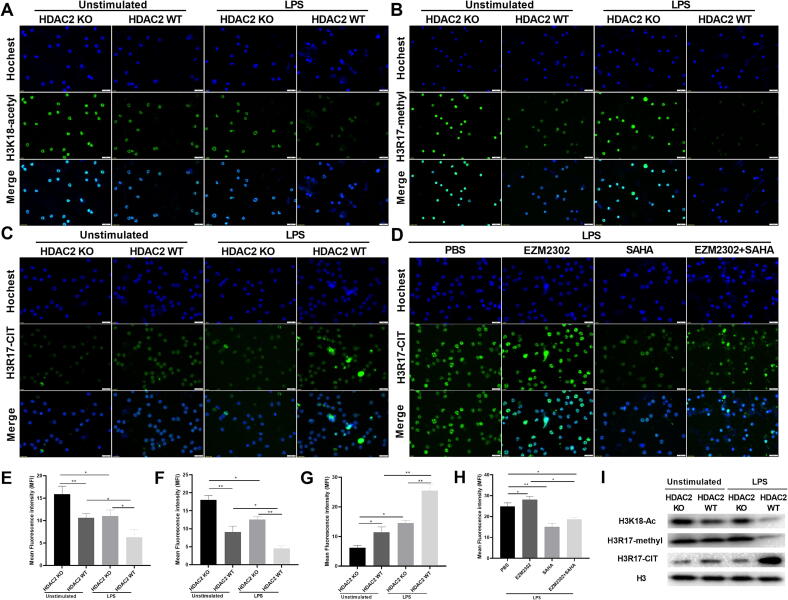
HDAC2 promotes NETs formation through the acetylation-citrullination pathway. (A–C) Representative images for LPS (50 μg/ml)-stimulated primary neutrophils from HDAC2 WT and HDAC2 KO mice. (A) Immunostaining for histone H3K18 acetylation (green) was performed with DNA counterstained with Hoechst 33342 (blue) and corresponding MFI of quantitative data (E). Scale bars, 20 μm. (B) Immunostaining for histone H3R17 methylation (green) was performed with DNA counterstained with Hoechst 33342 (blue) and corresponding MFI of quantitative data (F). Scale bars, 20 μm. (C) Immunostaining for histone H3R17 citrullination (green) was performed with DNA counterstained with Hoechst 33342 (blue) and corresponding MFI of quantitative data (G). Scale bars, 20 μm. (D) Histone H3R17 citrullination was regulated by H3R17 methylation inhibitor (EZM2302) and HDAC2 inhibitor (SAHA), and immunostaining for histone H3R17 citrullination (green) was performed with DNA counterstained with Hoechst 33342 (blue), the corresponding MFI of quantitative data (H). Scale bars, 20 μm. (I) H3K18 acetylation, H3R17 methylation and H3R17 citrullination were detected by western blotting in the neutrophils from HDAC2 WT and HDAC2 KO mice after stimulation with LPS (50 μg/ml). The H3 protein was used for western blot loading controls. (For interpretation of the references to colour in this figure legend, the reader is referred to the web version of this article.)

Previous studies have shown that histone citrullination is closely related to the formation of NETs [13]. To explore the role of HDAC2 in the regulation of histone citrullination, we detected the level of H3R17 citrullination in neutrophils by immunofluorescence. The results showed that LPS treatment significantly increased the level of H3R17 citrullination. In addition, the knockout or inhibition of HDAC2 could significantly impair the LPS-induced increases of H3R17 citrullination (Fig. 5C and D). Hence, it suggested that HDAC2 could potentially contribute to the NETs formation by facilitating histone citrullination at H3R17 site.

Studies have shown that histone modifying enzymes with opposing activities counteract each other’s effect through cross talks [25], and there exist dynamic changes in the modification patterns of histones between CARM1-mediated histone methylation and PAD4-mediated histone citrullination [13,25]. Additionally, several studies have revealed that HDAC2 regulates the H3K18 acetylation [26], and the histone H3K18 acetylation promotes CARM1-mediated methylation [27,28]. To further investigate the relationship among the HDAC2-mediated deacetylation, the CARM1-mediated methylation and the H3R17 citrullination, we detected the level of H3R17 methylation, H3K18 acetylation and H3R17 citrullination. The results showed that after LPS stimulation, the levels of H3K18 acetylation (Fig. 5A) and H3R17 methylation (Fig. 5B) in HDAC2 KO neutrophils were significantly higher than that of HDAC2 WT neutrophils. Western blot analysis corroborated the IF findings, confirming the same trend in H3K18 acetylation, H3R17 methylation and H3R17 citrullination levels (Fig. 5I). Furthermore, EZM2302, a methylation inhibitor targeting for CARM1, significantly decreased H3R17 methylation of neutrophil and reversed the inhibitory effect of the HDAC2 inhibitor on H3R17 citrullination induced by LPS (Fig. 5D and Supplementary Fig. 1). Altogether, these results suggested that HDAC2 restrained H3R17 methylation by downregulating the H3K18 acetylation in neutrophils. As a result, it promoted the H3R17 citrullination and ultimately contributed to the NETs formation.

### Dual strategy for HDAC2 and CARM1 inhibition improves the survival rate and organ dysfunction in CLP-induced septic mice

**Fig. 6:**
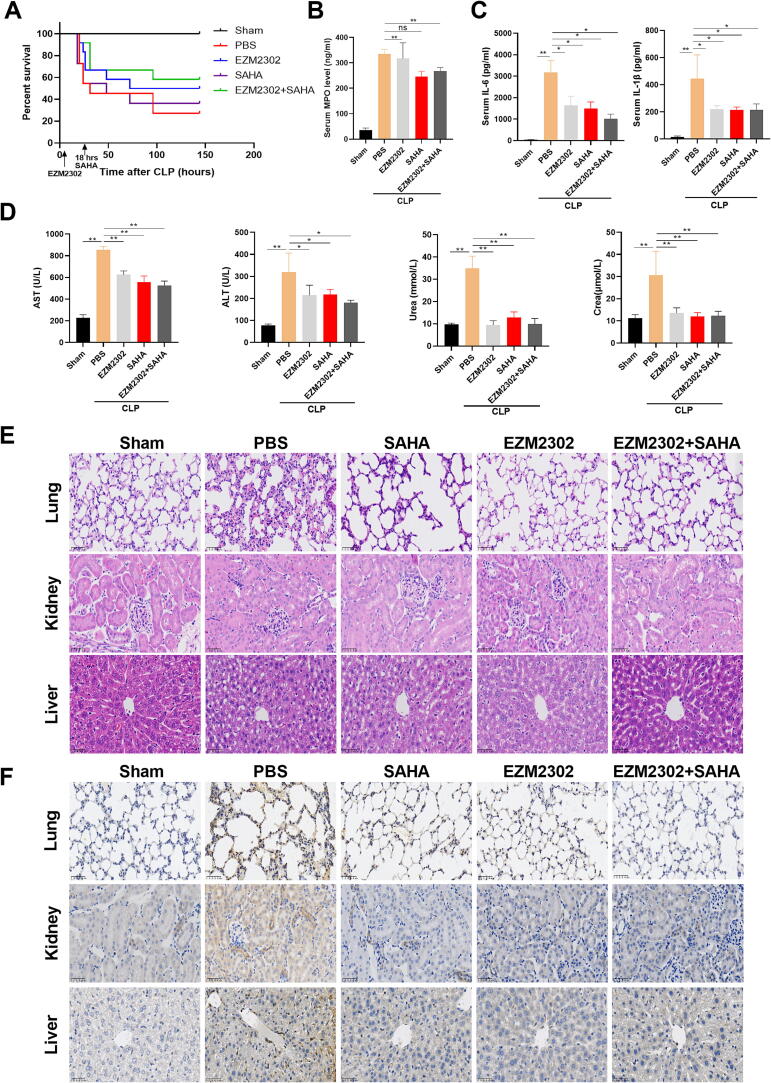
Protective effects of HDAC2 inhibition and histone methylation inhibition in CLP-induced septic mice (A) Kaplan-Meier curves of CLP-induced septic mice treated with or without SAHA and EZM2302 (n = 20 mice). (B and C) Serum MPO, IL-6 and IL-1β levels were measured with mouse ELISA kit in the CLP-induced septic mice treated with or without SAHA and EZM2302. (D) Serum AST, ALT, Urea and Crea levels were measured in the CLP-induced septic mice treated with or without SAHA and EZM2302. Blood was collected by orbital sampling, and serum biochemical parameters were measured by the Automatic Biochemistry Analyzer. The experiments were performed in quintuplicate, data are presented as the means ± SEM. of independent experiments. *P < 0.05, **P < 0.01 (two-tailed Student’s *t*-test). (n = 5 mice). (E and F) Histology of lungs, kidneys and livers from mice treated with or without SAHA and EZM2302. (E) The Histology with H&E staining for lungs, kidneys and livers. (F) Cell apoptosis in lungs, kidneys and livers was stained with cleaved-caspase 3. The mice were treated with EZM2302 (25 mg/kg) two hours prior to cecal ligation and puncture (CLP), and SAHA was administered after CLP surgery. SAHA, a HDAC2 inhibitor; EZM2302, a methylation inhibitor.

Although inhibition of HDAC2 alone reduced the inflammatory responses, it did not improve the survival rate of the infectious septic mice, due to the fact that it exacerbates the infection by reducing the NETs formation. Based on the above mechanism of HDAC2 promoting the NETs formation, we used both EZM2303 and SAHA to inhibit CARM1-mediated histone methylation and HDAC2-mediated histone deacetylation, respectively. The results showed that EZM2303 alone significantly increased the survival rate of CLP septic mice, improved their multiple organ dysfunction, including a significant reduction in blood biochemical indexes such as ALT, AST, urea and Cre, protected against the cell apoptosis of tissues in lung, kidney and liver, and slightly inhibited the inflammatory responses (Fig. 6A–E). In addition to significantly inhibiting the inflammatory responses, SAHA treatment alone only slightly improved the multiple organ dysfunction and protected against the cell apoptosis of tissues, but reduced the survival rate in CLP septic mice (Fig. 6B–E). However, combined treatment with EZM2303 and SAHA synergistically improved the survival rate, reduced the inflammatory responses, and protected against multiple organ dysfunction and cell apoptosis of tissues in CLP septic mice compared to EAM2303 and SAHA treatment alone (Fig. 6B–E). These results indicated that dual inhibition of HDAC2 and CARM1 may provide a brand-new idea for seeking effective ways to enhance not only antimicrobial but also anti-inflammatory activities in sepsis.

## Discussion

HDAC2, a member of the class I HDAC family, is an important epigenetic regulator that controls chromatin structure, transcription factor recruitment and gene expression by altering the histone acetylation at the promotor regions of targeting genes [27,29,30]. Although HDAC2 has been clinically shown to be closely associated with infection-related diseases, there are few mechanistic insights of HDAC2 regulating infection. At present, only a few studies have shown that HDAC2 is essential for the LPS-triggered inflammatory responses in macrophages [22]. LPS stimulation increases HDAC2 expression in macrophages. Knockout of HDAC2 results in the decreases in the expression of pro-inflammatory genes, such as IL-12, TNF-α and iNOS following stimulation with LPS [22]. As is known to all, sepsis is a life-threatening disease caused by infection which accompanies with uncontrolled inflammatory responses. Our study also found that HDAC2 was highly expressed in sepsis. Interestingly, knockout or inhibition of HDAC2 only enhanced the survival rate of the LPS-induced (non-infectious) septic mice, but reduced the survival rate of the CLP-induced (infectious) septic mice. Furthermore, this study demonstrated in the *E. coli* infection mouse model that the CFU counts in the kidney and blood were significantly higher in HDAC2 KO mice than in HDAC2 WT mice. Altogether, these results have indicated that HDAC2 not only regulates the inflammatory responses, but also plays an important role in the regulation of anti-infection immunity in sepsis. Therefore, although knockout or inhibition of HDAC2 can reduce the inflammatory responses, it also weakens the organism's anti-infection immunity and leads to the exacerbation of infection in septic mice.

Anti-infection therapy based on antibiotics is an indispensable means in the treatment of sepsis. However, the body's anti-infection immunity is also a key factor in the success of anti-infection treatment. Neutrophils are the first line of immune defense against infection in sepsis [[31], [32], [33]]. Neutrophil extracellular traps (NETs) are extracellular structures composed of a DNA backbone interspersed with histones, elastase, gelatinase, myeloperoxidases (MPO), and numerous antimicrobial proteins, its formation is a major mechanism by which neutrophils exert their anti-microbial effects [[31], [32], [33]]. These components are released by activated mature neutrophils in response to microorganisms, LPS, cytokines, or mitogens [34,35]. In this study, we have found for the first time that HDAC2 exerts the antimicrobial activities by promoting the formation of NETs, but not by affecting phagocytic uptake and killing capabilities of neutrophils (Fig. 4A–F). Previous evidences have shown that MPO and histone citrullination are two of the characteristics of NETs formation [36]. Our study has revealed that the level of MPO is notably decreased in neutrophils from HDAC2 KO mice following LPS treatment. Moreover, we also found that inhibition or knockout of HDAC2 significantly reduced the level of histone 3 citrullination of neutrophils. Our data suggest that HDAC2 is involved in the regulation of anti-infection immunity through promoting the formation of NETs. However, the mechanism by which HDAC2 promotes the formation of NETs is currently unclear.

Chromatin remodeling is a dynamic process that plays a crucial role in regulating cellular responses [37]. Previous studies have shown that chromatin swelling drives neutrophil extracellular trap and PAD4-mediated histone citrullination play a vital role in the regulation of NETs formation [9,31,35]. In addition, some studies have also shown that histone methylation is closely related to NETs formation through cross-talking with histone citrullination [13,25]. Our findings demonstrate that HDAC2 influences NETs formation through a cascade of histone modifications. Specifically, HDAC2 suppresses H3K18 acetylation, thereby attenuating the activity of CARM1-mediated H3R17 methylation. This attenuation ultimately upregulates H3R17 citrullination, promoting NETs formation. This sequence of events underscores the importance of chromatin remodeling in the regulation of NETs, a critical defense mechanism against bacterial infections. The spatial proximity of H3R17 and H3K18 is particularly noteworthy. These residues are located in close proximity on the histone tail, allowing for cross-talk between different histone modifications. Our study suggests that the interplay between acetylation at H3K18 and methylation at H3R17 is not merely coincidental but functionally interdependent. This interdependence highlights the complexity of chromatin remodeling and the potential for coordinated regulation of gene expression and cellular responses.

Many studies have shown that NETs are a double-edged sword in sepsis, involving the inflammatory responses, and bacterial killing and clearance [10]. Their excessive activation of NETs can lead to an inflammatory storm in the body, which may damage tissues and cause organ dysfunction [38]. Organ dysfunction is the main pathophysiological cause of sepsis and also a cause of the high mortality rate in sepsis. However, the deficiency of NETs formation leads to the worsen infection [[39], [40], [41]]. Our study has shown that besides upregulating inflammatory responses, HDAC2 has strong antimicrobial activities against bacterial infection by promoting the NETs formation. Thus, HDAC2 deficiency or inhibition can not only suppress excessive inflammatory responses but also restrain the NETs formation in septic mice. In order to eliminate the debilitating effect of HDAC2 inhibition on NETs formation, our study used a dual inhibition strategy of HDAC2 and CARM1 based on the previous discovery of the mechanism in this study, and the results showed that the dual inhibition strategy significantly improved the survival rate and multiple organ functions, and prevented from the cell apoptosis in tissues compared with SAHA or EZM2302 treatment alone in CLP-induced septic mice). Collectively, our study unveils a novel strategic approach for targeting sepsis with concurrent dual anti-inflammatory and antimicrobial outcomes, which is of great significance for the furtherance of effective strategies to treat sepsis.

Although our study provides great significance for the treatment of sepsis, there are still some questions that need to be solved in our study: Firstly, we have already found that HDAC2 upregulates the H3R17 citrullination. Current studies have shown that PAD4 mainly targets histone H3 R2/R8/R17 sites in citrullination. On this ground, whether HDAC2 functions on other sites of histone H3 is still a question that researchers need to continue to explore in future; Secondly, we have realized that the lifespan of stimulant-induced activated neutrophils is only 4–8 h, which makes it much more difficult to verify the effect of histone H3R17 citrullination on the formation of NETs through the means of gene knockout in primary neutrophils, which is also a large challenge for future research on antimicrobial and anti-inflammatory immunity. Finally, we have discovered that the occurrence of NETs is regulated by ROS, and we have also observed a significant reduction in ROS and NETs production following HDAC2 deficiency. Nevertheless, the specific mechanism underlying this observation remains elusive and warrants further investigation.

## Conclusion

This study has revealed that HDAC2 enhances the antimicrobial activities by promoting the formation of NETs for the first time. Mechanistically, HDAC2 indirectly promotes the histone H3R17 citrullination to induce the NETs formation through downregulating H3K18 acetylation and interactively inhibiting H3R17 methylation in neutrophils. Furthermore, dual inhibition strategy for HDAC2-mediated acetylation and CARM1-mediated methylation might provide a novel therapeutic strategy to prevent or treat sepsis by enhanced antimicrobial and anti-inflammatory properties. Hence, our study provides novel leads and directions for scientific researchers to delve deeper into molecular regulatory mechanisms and strategies by which HDAC2 orchestrates the treatment of sepsis through enhancing the NETs formation and anti-inflammatory responses.

## Compliance with ethics requirements

The mice were housed at a controlled temperature of 22–24 °C with 12 h light–dark cycles. The mice were offered standard laboratory tap water and chow ad libitum. All applicable international and institutional guidelines for the care and use of animals were followed. The mice experiments were performed according to the protocols approved by the Ethics Review Committee for Animal Experimentation of Daping Hospital, Army Medical University. All mice were treated humanely throughout the experimental period.

## Author contributions

Xiang Xu, Huaping Liang and Sai Wang were responsible for the conceptualization and planning of the study; Zhan Li, Kaiyan Lv, Wang Hu, Lumin Sui, Mu Yuan, Luoquan Ao, Quan Chen, Junxia Li and Lixing Tian performed the research; Zhengbi Liu and Sai Wang were responsible for the care and breeding of laboratory mice; Xiang Xu, Zhan Li, Wang Hu and Kaiyan Lv were in charge of data analysis and manuscript preparation. All authors have reviewed and endorsed the final manuscript.

## Ethics approval and consent to participate

All applicable international and institutional guidelines for the care and use of animals were followed. The mice experiments were performed according to the protocols approved by the Ethics Review Committee for Animal Experimentation of Daping Hospital, Army Medical University (No. SYXK20170002). All mice were treated humanely throughout the experimental period.

## Declaration of competing interest

The authors declare that they have no known competing financial interests or personal relationships that could have appeared to influence the work reported in this paper.

## Figures and Tables

**Supplementary Fig. 1:**